# Dissemination of Chronic Disease Self-Management Education (CDSME) Programs in the United States: Intervention Delivery by Rurality

**DOI:** 10.3390/ijerph14060638

**Published:** 2017-06-14

**Authors:** Matthew Lee Smith, Samuel D. Towne, Angelica Herrera-Venson, Kathleen Cameron, Kristie P. Kulinski, Kate Lorig, Scott A. Horel, Marcia G. Ory

**Affiliations:** 1 College of Public Health, The University of Georgia, Athens, GA 30602, USA; 2 School of Public Health, Texas A&M University, College Station, TX 77844, USA; towne@sph.tamhsc.edu (S.D.T.J.); sahorel@sph.tamhsc.edu (S.A.H.); mory@sph.tamhsc.edu (M.G.O.); 3 National Council on Aging, Arlington, VA 22202, USA; angelica.herrera-venson@ncoa.org (A.H.-V.); kathleen.cameron@ncoa.org (K.C.); 4 Administration for Community Living, Washington, DC 20201, USA; Kristie.Kulinski@acl.hhs.gov; 5 School of Medicine, Stanford University, Palo Alto, CA 94304, USA; lorig@stanford.edu

**Keywords:** rural, chronic disease, self-management, evidence-based program, dissemination, implementation, United States of America

## Abstract

*Background*: Alongside the dramatic increase of older adults in the United States (U.S.), it is projected that the aging population residing in rural areas will continue to grow. As the prevalence of chronic diseases and multiple chronic conditions among adults continues to rise, there is additional need for evidence-based interventions to assist the aging population to improve lifestyle behaviors, and self-manage their chronic conditions. The purpose of this descriptive study was to identify the geospatial dissemination of Chronic Disease Self-Management Education (CDSME) Programs across the U.S. in terms of participants enrolled, workshops delivered, and counties reached. These dissemination characteristics were compared across rurality designations (i.e., metro areas; non-metro areas adjacent to metro areas, and non-metro areas not adjacent to metro areas). *Methods*: This descriptive study analyzed data from a national repository including efforts from 83 grantees spanning 47 states from December 2009 to December 2016. Counts were tabulated and averages were calculated. *Results*: CDSME Program workshops were delivered in 56.4% of all U.S. counties one or more times during the study period. Of the counties where a workshop was conducted, 50.5% were delivered in non-metro areas. Of the 300,640 participants enrolled in CDSME Programs, 12% attended workshops in non-metro adjacent areas, and 7% attended workshops in non-metro non-adjacent areas. The majority of workshops were delivered in healthcare organizations, senior centers/Area Agencies on Aging, and residential facilities. On average, participants residing in non-metro areas had better workshop attendance and retention rates compared to participants in metro areas. *Conclusions*: Findings highlight the established role of traditional organizations/entities within the aging services network, to reach remote areas and serve diverse participants (e.g., senior centers). To facilitate growth in rural areas, technical assistance will be needed. Additional efforts are needed to bolster partnerships (e.g., sharing resources and knowledge), marketing (e.g., tailored material), and regular communication among stakeholders.

## 1. Introduction

As the population in the United States (U.S.) ages, the number of older adults (aged 65 years and older) residing in rural areas is also growing [1]. Some estimates suggest that if current trends continue, rural areas will see a 30% growth in those aged 55–75 years in the decade leading up to 2020 [2]. In 2014, there were over 46 million older adults residing in the U.S., which represented about 14.5% of the population [3]. By 2060, the older adult population is expected to double in size, with about 98 million older Americans [3]. As Americans live longer, their risk for acquiring multiple chronic conditions increases. An estimated 25% of adults aged 18 and over have two or more chronic conditions, and this estimate increases to approximately 67% among older adults [4,5]. Common chronic conditions among older adults include cardiovascular disease, diabetes, arthritis, cancer, lung disease, and depression. The burden of chronic conditions accounts for the majority of the healthcare budget in the United States [4], and has long-lasting impacts on the physical and mental health of older adults and their caregivers/support systems [6].

Aging may be seen as a unique rural phenomenon in that migration to non-metro (i.e., small cities and rural areas) has been shown to be associated with increasing age, in part, due to being seen by some as retirement destinations [2]. An estimated 20% of Americans reside in rural areas, which represents over 80% of the U.S. land area [7]. Approximately 19% of older adults reside in rural areas, and an additional 16% reside in micropolitan areas (urban clusters with more than 10,000 residents, but fewer than 50,000 residents) [8]. The context of living in rural areas presents unique health challenges to older adults [9]. More specifically, older adults in rural areas have higher rates of obesity [10], more complex disease profiles, experience isolation [11,12], and have limited access to healthcare and health-related resources and services [13,14]. Health-related service utilization is limited among older adults in rural areas in part because of their geographic proximity to health-providing agencies and resources [15,16]. Travel time to obtain health-related services is increased in many rural communities, because service providers are less abundant, and more dispersed over larger land areas [15]. As such, it is critical that service provision in rural areas be strategically allocated to increase availability and accessibility [9]. Further, health disparities go beyond a simple rural–urban dichotomy into social and structural determinants of health [17]. For example, factors associated with health and health-related factors (e.g., access to care) may include structural (e.g., public policy) and social determinants of health (e.g., income, education) [17]. In rural areas, health disparities may be affected by a host of issues including, but not limited to, education, income, and race/ethnicity [18,19]. Furthermore, health literacy can also contribute to poor health-related outcomes, and at the same time, health literacy may be lower in rural areas [20] which could lead to further health disparities. Thus, the need to target the underlying mechanisms of poor health and related outcomes is crucial. In light of these barriers to health service use, older adults residing in rural areas may gain the most benefit from chronic disease self-management interventions, that both empower them to make positive lifestyle changes, and prevent disease-related complications associated with costly care and further debilitation. 

The growing emphasis on community-based interventions to promote healthy aging reflects the growing recognition, that older adults with chronic conditions spend only a small proportion of time in healthcare settings (e.g., physician visits), and are responsible for the day-to-day management of their conditions. Further, this emphasis is indicative of the U.S. evolving from a paternalistic healthcare system, to one that provides more autonomy and empowerment to older adults, with the support of healthcare providers and services. To combat the impact and consequences of chronic disease among older adults, numerous evidence-based programs have been developed and disseminated to improve lifestyle and self-management behaviors. The Chronic Disease Self-Management Education (CDSME) Programs are among the best known and most widely disseminated [21,22]. Created by the Patient Education Research Center at Stanford University, CDSME Programs are a suite of interventions rooted by the Social Learning Theory [23] and offered in small groups. 

The flagship CDSME Program is the Chronic Disease Self-Management Program (CDSMP). This process-based intervention is universal for people living with any/all chronic conditions and uses problem solving, action planning, and goal setting to build participants’ skills in managing their disease symptomology. Examples of topics discussed during the workshops include physical activity, nutrition, techniques to cope with health problems and negative emotions, communication with healthcare providers, and how to assess new treatments. The effectiveness of CDSMP was initially documented from a randomized-controlled trial conducted in the late 1990s [24,25,26]. Over the past two decades, the program has been translated for community use and widely disseminated through a national training, certification and licensure infrastructure. As documented in the *National Study of CDSMP*, the grand-scale dissemination of the intervention maintained its effectiveness in terms of symptom management, physical and mental health outcomes, and self-reported healthcare utilization reductions (i.e., hospitalizations, emergency room visits) [27,28,29]. 

In addition to being universally appropriate for any chronic condition, CDSMP has been developed to address specific conditions (e.g., diabetes, arthritis, chronic pain, cancer). These generic and condition-specific CDSMP versions comprise the suite of CDSME Programs. Each CDSME Program workshop is facilitated by two leaders who have undergone rigorous training. Detailed manuals exist for facilitation, implementation, and fidelity. Each small-group workshop consists of six sessions, each of which last 2.5 h. Workshop sessions take place once a week for six consecutive weeks. CDSME Programs are primarily offered in English and Spanish, although they are available in approximately 17 languages.

In the U.S., the Administration for Community Living/Administration on Aging (ACL/AoA) has provided discretionary grants programs to support evidence-based health programs. From 2006 to 2016, there have been four main waves of funding to promote the dissemination of interventions to vulnerable Americans, defined as those who are underserved and in the greatest need for services (e.g., racial/ethnic minorities; low socioeconomic status; living in geographically underserved areas) [30,31]. In a study by Towne et al. (2015), which examined the first 100,000 participants reached by CDSMP as part of the ACL/AoA initiative, it was determined that about half of U.S. counties were reached by the intervention [32]. However, approximately 75% of the counties without CDSMP were designated as rural, highlighting an implementation disparity [32]. While Towne et al. (2015) examined the rural reach of CDSMP, their study focused on data pertaining to residential residence, not the workshop delivery locations. Further, it did not examine all programs in the expanding CDSME Program suite. In contrast, the current study used programmatic and organizational data to examine the nuances of CDSME Program delivery in rural areas. 

The primary purpose of this descriptive study was to identify the geospatial dissemination of CDSME Programs across the U.S. in terms of participants enrolled, workshops delivered, and counties reached. Dissemination characteristics were compared across rurality designations, to examine program reach to serve vulnerable individuals. Secondarily, data were also stratified by CDSME Program workshop and delivery site-type, in order to facilitate a more detailed assessment of characteristics related to the participants, delivery sites, and workshops. These characteristics were examined to provide contextual information about this multi-year national dissemination effort. 

## 2. Materials and Methods

### 2.1. Study Participants and Procedures

Data for this study utilized a national data repository created alongside a series of national funding initiatives to support the dissemination of CDSME Programs as part of the American Recovery and Reinvestment Act of 2009 Communities Putting Prevention to Work: CDSMP initiative [31,33]. Kulinski et al. (2015) provide an in-depth account of the rationale for selecting standardized measures, as well as data collection tools, coordination, and processes used to develop and operate this national, online database [31]. Data components in the data repository include workshop information, participant information, workshop attendance records, and organization data. These data types are collected locally by workshop leaders and organizations hosting programs. Data can be entered in a centralized or de-centralized manner at the state or regional level. Data used for this study included efforts from 83 grantees spanning 47 states from December 2009 to December 2016. It is important to note that ACL/AoA grantees are required to use this national data repository. However, data entered in the system does not necessarily represent all of the CDSME Program delivery across the United States (i.e., other areas and organizations are offering CDSME Programs, but are not required to use the repository). Institutional Review Board approval was granted from The University of Georgia (#00000249) for this secondary, de-identified data analyses.

### 2.2. Data and Measures

#### 2.2.1. Dependent Variable

The dependent variable for this descriptive study was CDSME Programs delivery in rural areas. To categorize counties, each was assigned a Rural-Urban Continuum Code (RUCC). RUCCs were developed by the United States Department of Agriculture and use a 9-point scale to indicate proximity to nearby metro areas [34,35,36]. For this study, three categories were created to document rurality in terms of adjacency to metro areas: metro areas (i.e., RUCC of 1, 2, and 3); non-metro adjacent areas (i.e., RUCC of 4, 6, and 8), and non-metro non-adjacent areas (i.e., RUCC of 5, 7, and 9). More information about each of the nine RUCC classifications and associated methodology can be found elsewhere [37]. Among non-metro areas, the separating relative adjacency to metropolitan areas versus not adjacent to metro areas, which may matter in terms of access to care [38], allowed for assessing more than a simple rural-urban dichotomy.

#### 2.2.2. Workshop and County Characteristics

Using data in the national repository, the total number of participants enrolled, workshops delivered, and unique counties reached were tabulated. Based on the ZIP Code of the workshop delivery location, the ZIP Code Tabulation Area (ZCTA) was identified, and population statistics were obtained. More specifically, the ZCTA of the workshop delivery location was used to identify the median household income (i.e., 2011 inflation-adjusted dollars), percent of residents residing in poverty (i.e., ratio of income to poverty level in the past 12 months), percent of residents who were white, percent of residents who were Hispanic, and percent of residents with less than a high school education. The average number of participants enrolled in each workshop was calculated. The average number of workshop sessions attended was reported (i.e., ranging from 1 to 6 sessions). Additionally, the proportion of participants that completed workshops (i.e., attending 4+ of the 6 offered sessions) was calculated [39]. 

#### 2.2.3. Workshop Type

Because the CDSME Program suite consists of multiple tailored interventions, the dissemination of each unique workshop type was of interest. English-language workshops included: Arthritis Self-Management Program (ASMP); Chronic Disease Self-Management Program (CDSMP); Diabetes Self-Management Program (DSMP); Chronic Pain Self-Management Program (CPSMP); Positive Self-Management Program (PSMP), and Cancer: Thriving and Surviving (CTS) [40]. Spanish-language workshops included: Spanish ASMP; Tomando Control de su Salud (Spanish CDSMP); and Programa de Manejo Personal de la Diabetes (Spanish DSMP) [41]. Additional English-language workshops included HomeMeds [42]. Other workshop types and those not identified in the database were also reported.

#### 2.2.4. Delivery Site Type

Given the diverse organizational infrastructure used to deliver CDSME Programs, the delivery site type for workshops was of interest. Delivery site types included: healthcare organizations; senior centers/Area Agencies on Aging (AAA); residential facilities; multi-purpose organizations (including parks and recreation facilities, and libraries); faith-based organizations; educational institutions; county health departments; workplaces; tribal centers; and other site types [43,44].

#### 2.2.5. Sociodemographics

Personal characteristics of the participants enrolled in CDSME Programs included age, gender, and number of self-reported chronic conditions.

### 2.3. Statistical Methods

**Figure 1:**
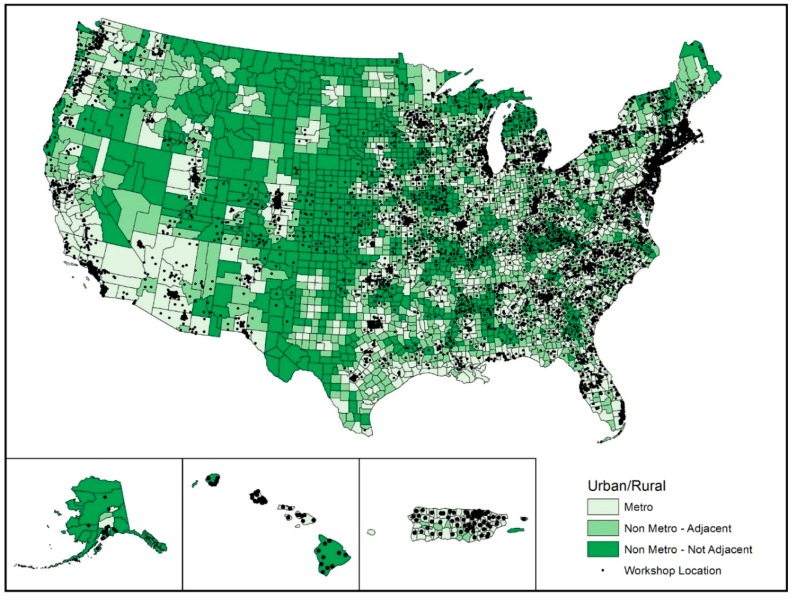
Geospatial distribution of Chronic Disease Self-Management Education (CDSME) Program workshops by county rurality.

All analyses were performed using SPSS (version 24, IBM Corporation, Chicago, IL, USA) for this descriptive study. Counts were tabulated for the number of participants enrolled, number of workshops delivered, and number of unique counties reached. These counts were stratified by workshop type and rurality (see Table 1). Counts were also stratified by delivery site type and rurality (see Table 2). Averages were calculated for participant characteristics, workshop delivery location characteristics (yielded from ZCTA of the workshop location), and workshop characteristics. ArcGIS (Environmental Systems Research Institute, Redlands, CA, USA) was used to geospatially map the delivery location of each workshop (see Figure 1).

## 3. Results

Figure 1 illustrates the national delivery of CDSME Programs by rurality. Darker shading indicates counties classified as more rural. Black dots indicate the physical location of CDSME Program delivery sites. Figure 1 is supported by data provided in Table 1, which describes the delivery of individual CDSME Programs by rurality in terms of the number of participants enrolled, number of workshops delivered, and number of counties reached. 

Overall, 300,640 participants were enrolled in CDSME Programs over the study period. The majority of participants attended workshops in metro areas (82%), followed by workshops in non-metro adjacent areas (12%) and non-metro non-adjacent areas (7%). Overall, 25,279 workshops were delivered over the study period. The majority of workshops were delivered in metro areas (80.5%), followed by workshops in non-metro adjacent areas (11.5%) and non-metro non-adjacent areas (8%). When examining CDSME Program delivery by workshop type, CDSMP was the most widely disseminated (210,587 participants; 17,999 workshops; and 1408 counties), followed by DSMP (51,647 participants; 4476 workshops; and 283 counties), Spanish CDSMP (21,945 participants; 1656 workshops; and 68 counties), and CPSMP (6287 participants, 551 workshops, and 30 counties). In the U.S., there are 3221 counties [45]. CDSME Program workshops were delivered in 1818 counties (56.4%), one or more times, from December 2009 to December 2016. Of the counties where a workshop was conducted, 50.5% were delivered in non-metro areas. Workshops were delivered in 899 of the 1236 metro counties (72.7%), 504 of the 1034 non-metro adjacent counties (48.7%), and 415 of the 951 non-metro non-adjacent counties (43.6%). 

Table 2 describes the delivery of CDSME Programs by delivery site-type and rurality, in terms of the number of participants enrolled, number of workshops delivered, and number of counties reached. The five most prevalent delivery site types were healthcare organizations (73,646 participants; 6551 workshops; and 311 counties), senior centers/AAA (71,052 participants; 5900 workshops; and 577 counties), residential facilities (52,395 participants; 4230 workshops; and 266 counties), multi-purpose organizations (34,498 participants; 3029 workshops; and 258 counties), and faith-based organizations (24,177 participants; 1977 workshops; and 144 counties). Despite workshops delivered in healthcare organizations enrolling more participants and holding more workshops, workshops delivered in senior centers/AAA reached more counties (i.e., 577 versus 311). While CDSME Programs were most prevalent in metro areas, workshops delivered in educational institutions, workplaces, and tribal centers were more prevalent in rural areas (i.e., more participants enrolled, more workshops delivered, and more counties reached in non-metro non-adjacent areas relative to non-metro adjacent areas).

Table 3 describes the participant, delivery site location, and workshop characteristics by rurality. Overall, the average participant age was 65.40 (±15.28) years, and participants reported an average of 2.06 (±1.66) chronic conditions. On average, workshops were delivered in areas with median household incomes of $49,299.18 (±$21,019.93); 18.48% (±11.73%) of residents living in poverty; 69.05% (±24.91%) of residents being white; 16.64% (±21.58%) of residents being Hispanic; and 17.15% (±10.89%) of participants having less than a high school education. Compared to workshops delivered in metro areas, workshops delivered in non-metro non-adjacent areas enrolled younger participants (i.e., average age of 63.99 compared to 65.76 years). Compared to workshops delivered in metro areas, workshops delivered in non-metro non-adjacent areas had lower median household incomes (i.e., average income of $39,771.14 compared to $51,257.75), had more white residents (i.e., 83.19% compared to 66.50%), and had fewer Hispanic residents (i.e., 6.74% compared to 18.78%).

On average, CDSME Program workshops enrolled 13.50 (±6.32) participants, of which 9.49 (±4.47) completed the workshops (i.e., attended 4+ of the 6 sessions). On average, participants attended 4.31 (±1.75) workshop sessions. Compared to workshops delivered in metro areas, workshops delivered in non-metro non-adjacent areas enrolled fewer participants (i.e., 12.09 compared to 13.77 participants enrolled), but had higher overall attendance (i.e., 4.46 compared to 4.27 sessions attended).

## 4. Discussion

This study provides a unique examination of a national dissemination of a set of evidence-based disease self-management interventions targeted toward older adults. Intervention penetration is an important issue in making a visible impact on population health [46]. Over half of United States counties were reached by CDSME Program workshops (56.4%). However, the majority of workshops delivered (80.4%) and participants enrolled (82.1%) were in metro areas. Further, the proportion of metro counties served was greater than non-metro counties nationwide (i.e., 72.7% in metro areas, relative to 48.7% in non-metro adjacent, and 43.6% in non-metro non-adjacent areas). These findings confirm the relative lack of access to health-related resources in rural counties [47]. Therefore, despite the potential reach of these interventions in rural areas, additional efforts are needed to ensure adequate workshop delivery to serve populations in rural communities [32]. 

Despite an attempt to reach more ethnically diverse older adults, the Spanish-language version of CDSMP was not as widely disseminated as the English-language version. Spanish-language workshops, and Hispanic residents, were heavily concentrated in metro regions. It was also observed that some workshop types were more prevalent in certain geographic settings. For example, CDSMP was predominant in non-metro counties (i.e., 641 in metro counties, relative to 418 in non-metro adjacent, and 349 in non-metro non-adjacent areas), in terms of a simple comparison of the number of counties reached. However, rural areas are most at-risk in terms of the proportions (i.e., smaller proportion of rural counties served, relative to metro areas). The presence and expansion of CDSMP was not surprising, and this is likely testament to its longer history and record of financial and community support (relative to other CDSME Program workshop types, which are now gaining traction). When introduced into a community, ample time is needed to develop a training and delivery infrastructure, capable of hosting ongoing workshops that will serve a sufficient number of individuals with chronic conditions residing in a particular area. Furthermore, host/sponsoring agencies (e.g., Area Agencies on Aging) located in rural areas, or those delivering programing in rural areas, may have greater distances to travel, with higher associated travel costs (i.e., longer distances lead to higher cost of gas and potential reimbursement for employees) [48]. In terms of funding, adjustment for this added cost is crucial when planning resource allocation. Tailored marketing material, with distinct target populations in mind, can be another helpful addition in recruitment and retention of participants [48]. Additionally, based on the successes of delivering disease self-management programs in online formats [49,50,51], the use of internet-delivered interventions should also be considered to increase intervention access in remote areas. 

In previous studies examining the national roll-out of CDSME Programs, the majority of participants were enrolled in workshops in senior centers/AAA [43,52]. However, in this study, examining the maturation of this nationwide dissemination effort, healthcare organizations have emerged as the delivery site-type delivering the largest number of workshops and enrolling the largest number of participates. However, it should be noted that senior centers/AAA reached many more counties (*n* = 577), relative to healthcare organizations (*n* = 311), over half of which were in non-metro areas (*n* = 305). These findings highlight the established role of traditional organizations/entities within the aging services network, to serve remote areas and diverse participants, within the reach of 11,000 senior centers nationwide. Meanwhile, the growth of healthcare organizations as primary delivery sites denotes their organizational alignment with the intervention itself, as delivery settings able to identify and enroll patients with chronic conditions. The rise of healthcare organizations as primary delivery sites in this national dissemination shows the success in diversifying the delivery infrastructure to engage primary care practices, insurance carriers, and other healthcare payers, as a means of reaching patients with more complex disease profiles, and sustaining CDSME Program workshop offerings in communities across the country. The latter is also in line with recent grant announcements, highlighting the need to create innovative pay structures and incentives for scalability and sustainability [53].

When examining the delivery sites offering CDSME Program workshops, educational institutions, workplaces, and tribal centers were able to enroll more participants, deliver more workshops, and reach more counties in the most rural of settings (i.e., non-metro non-adjacent, relative to non-metro adjacent). Despite less enrollment, delivery, and reach, relative to non-metro adjacent areas, other delivery site types had comparable dissemination efforts in the most rural settings (e.g., senior centers/AAA, county health departments, faith-based organizations). This signifies the strong role of these organizations/entities in more rural areas, and their established community trust, and their function as places for congregation [48,54]. Based on the success of these delivery sites, future efforts are needed to engage such entities in rural communities, in order to adopt CDSME Programs and routinely offer them to local residents. To facilitate growth in rural areas, technical assistance will be needed (e.g., topics related to delivery structure enhancement, training and retaining lay leaders, participant recruitment, transportation). Previous experience by the study team highlights the utility of partnerships (e.g., sharing resources and knowledge), marketing (e.g., tailored material), and regular communication among stakeholders (e.g., among Area Agencies on Aging and program evaluators) [48]. While this study examined the implementation of programs within a larger national dissemination effort, it did not specifically assess rural reach, by stratifying each individual program by the delivery site types hosting workshops. While organizational adoption and delivery characteristics differences are assumed between CDSME Programs, such an examination is beyond the scope of this study. Future studies should examine the nuances of each CDSME Program dissemination, especially by the most predominant programs and delivery sites. 

Relative to workshops delivered in metro areas, those offered in non-metro non-adjacent areas had larger proportions of families living below the poverty line. This finding highlights socioeconomic vulnerability in these communities, which are therefore, areas that can benefit most from low-cost and/or free resources. While rural areas had smaller workshop sizes in terms of participants enrolled, their completion rates (i.e., those attending 4+ of 6 sessions) were higher than those for workshops hosted in metro areas. One explanation of this finding may be based on the community dynamics (e.g., higher social cohesion) within rural communities [55]. Another explanation may be that carpooling or other forms of coordinated transport took place, to minimize the transportation deficiencies and travel burdens characteristic in rural communities [48,56]. Therefore, it is recommended that CDSME Program workshops be offered through a more diversified delivery infrastructure, encompassing locations closer to participants’ homes (to minimize travel burden to attend 6-week CDSME Program workshops) [57]. The latter changes may further bolster recruitment and retention in rural areas.

This study is not without limitations. First, there are many forms of rural designations, which have been created for a variety of reasons [58]. It is acknowledged that the designation used in this study was selected based on proximity to urban areas, but other designations may have yielded different and meaningful information. Future studies should replicate the study methodology to examine the implications of applying other rural criteria (e.g., Health Provider Shortage Areas, Medically Underserved Areas). This study used geospatial mapping to illustrate the delivery of CDSME Programs across the United States by rurality. However, future efforts should strive to add additional map layers to examine other community characteristics in relation to workshop delivery (e.g., unengaged organizations that could host programs, disease-related “hot-spots”) [59]. Lastly, as mentioned previously, this dataset primarily consisted of data from ACL/AoA grantees, and is thus it not fully representative of the ongoing CDSME Program dissemination nationwide (~50% to 75% of all CDSME Program activity). Additionally, it should be noted that CTS was released for use only about one year ago, and ASMP is in the process of being ‘phased out’ and replaced by CDSMP and PSMP. 

## 5. Conclusions

Overall, large gaps in availability of programing were seen in the Midwest and other areas through the U.S., with gaps in programing primarily affecting rural areas. Given the already present gaps in access to care in these regions [32,46,60], policy makers and other decision makers should consider the unique needs of those in rural areas when allocating resources (e.g., funding/reimbursement for delivery). While significant, yet relatively limited, reach in rural areas has been seen, much more is needed in order to match that of the proportion reached in metropolitan areas. Continued monitoring of trends in program delivery are recommended to assess whether many of the nations’ potentially vulnerable populations have access to these potentially life-changing programs. 

## Figures and Tables

**Table 1:** CDSME Program workshop types by rurality.

Workshop Type	Participants				Workshops (Counties)			
	Metro	Non-Metro: Adjacent	Non-Metro: Not Adjacent	Total	Metro	Non-Metro: Adjacent	Non-Metro: Not Adjacent	Total
ENGLISH-LANGUAGE WORKSHOPS								
Arthritis Self-Management Program (ASMP)	651	168	43	862	51 (3)	11 (3)	4 (0)	66 (6)
Chronic Disease Self-Management Program (CDSMP)	166,936	26,002	17,649	210,587	13,964 (641)	2344 (418)	1691 (349)	17,999 (1408)
Diabetes Self-Management Program (DSMP)	44,735	4384	2528	51,647	3843 (165)	397 (67)	236 (51)	4476 (283)
Chronic Pain Self-Management Program (CPSMP)	4928	802	557	6287	419 (16)	75 (8)	57 (6)	551 (30)
Positive Self-Management Program (PSMP)	98	9	0	107	13 (0)	1 (0)	0 (0)	14 (0)
Cancer: Thriving and Surviving (CTS) (English)	314	0	0	314	30 (1)	0 (0)	0 (0)	30 (1)
SPANISH-LANGUAGE WORKSHOPS								
Spanish ASMP	87	0	0	87	1 (0)	0 (0)	0 (0)	1 (0)
Tomando Control de su Salud (Spanish CDSMP)	20,574	726	645	21,945	1536 (53)	61 (8)	59 (7)	1656 (68)
Programa de Manejo Personal de la Diabetes (Spanish DSMP)	4571	117	30	4718	382 (18)	8 (0)	4 (0)	394 (18)
ADDITIONAL WORKSHOPS								
HomeMeds (English)	3999	0	0	3999	82 (2)	0 (0)	0 (0)	82 (2)
Other/Not Identified	60	19	8	87	6 (0)	2 (0)	2 (2)	10 (2)
TOTAL	246,953	32,227	21,460	300,640	20,327 (899)	2899 (504)	2053 (415)	25,279 (1818)

(0) = no unique county served with this program.

**Table 2:** Delivery site types by rurality.

Delivery Site Type	Participants				Workshops (Counties)			
	Metro	Non-Metro: Adjacent	Non-Metro: Not Adjacent	Total	Metro	Non-Metro: Adjacent	Non-Metro: Not Adjacent	Total
Healthcare Organization	62,294	6507	4845	73,646	5383 (159)	666 (86)	502 (66)	6551 (311)
Senior Center/Area Agency on Aging	56,796	8381	5875	71,052	4658 (272)	711 (155)	531 (150)	5900 (577)
Residential Facility	45,785	4137	2473	52,395	3637 (147)	368 (75)	225 (44)	4230 (266)
Multi-Purpose Organization/Parks & Rec/Library	29,100	3589	1809	34,498	2491 (139)	344 (68)	194 (51)	3029 (258)
Faith-Based Organization	19,825	2377	1975	24,177	1600 (60)	207 (48)	170 (36)	1977 (144)
Educational Institution	4811	305	868	5984	384 (24)	33 (9)	73 (14)	490 (47)
County health department	2771	958	402	4131	292 (17)	106 (13)	55 (12)	453 (42)
Workplace	1752	85	406	2243	166 (6)	10 (1)	43 (4)	219 (11)
Tribal center	618	38	125	781	56 (3)	6 (3)	13 (3)	75 (9)
Other	23,201	5850	2682	31,733	1660 (72)	448 (46)	247 (35)	2355 (153)
Total	246,953	32,227	21,460	300,640	20,327 (899)	2899 (504)	2053 (415)	25,279 (1818)

**Table 3:** Participant, delivery site location, and workshop characteristics by rurality.

Variables	Total	Metro	Non-Metro: Adjacent	Non-Metro: Not Adjacent
Age	65.40 (±15.28)	65.76 (±15.03)	63.58 (±16.50)	63.99 (±15.99)
Number of Chronic Conditions	2.06 (±1.66)	2.06 (±1.65)	2.09 (±1.68)	2.05 (±1.71)
Median Household Income *	$49,299.18 (±$21,019.93)	$51,257.75 (±$22,156.63)	$40,743.13 (±$11,699.29)	$39,771.14 (±$9723.27)
Percent Living Over Poverty Line *	18.48% (±11.73%)	18.36% (±12.33%)	19.18% (±8.85%)	18.78% (±7.83%)
Percent White *	69.05% (±24.91%)	66.50% (±24.86%)	79.17% (±22.36%)	83.19% (±20.02%)
Percent Hispanic *	16.64% (±21.58%)	18.78% (±22.66%)	6.90% (±11.41%)	6.74% (±11.41%)
Percent Less than High School Education *	17.15% (±10.89%)	17.12% (±11.37%)	17.46% (±8.39%)	16.94% (±8.22%)
Number of Participants Enrolled in Workshops	13.50 (±6.32)	13.77 (±6.58)	12.43 (±4.75)	12.09 (±4.80)
Number of Participants Completing Workshops (attend 4+ of 6)	9.49 (±4.47)	9.54 (±4.48)	9.41 (±4.49)	9.11 (±4.32)
Number of Sessions Attended (of 6)	4.31 (±1.75)	4.27 (±1.76)	4.46 (±1.71)	4.46 (±1.69)

* Indicates statistic from the ZIP Code Tabulation Area (ZCTA) of the delivery site location.
